# 7-Dimethyl­amino-2-phenyl-1,2,4-triazolo[1,5-*a*][1,3,5]triazin-5-amine methanol solvate^1^

**DOI:** 10.1107/S1600536808030481

**Published:** 2008-09-27

**Authors:** Anton V. Dolzhenko, Geok Kheng Tan, Lip Lin Koh, Su Fen Woo, Wai Keung Chui

**Affiliations:** a Department of Pharmacy, Faculty of Science, National University of Singapore, 18 Science Drive 4, Singapore 117543, Singapore; b Department of Chemistry, Faculty of Science, National University of Singapore, 3 Science Drive 3, Singapore 117543, Singapore

## Abstract

7-Dimethyl­amino-2-phenyl-1,2,4-triazolo[1,5-*a*][1,3,5]triazin-5-amine crystallized with one mol­ecule of methanol to give the title compound, C_12_H_13_N_7_·CH_3_OH. The triazolo[1,5-*a*][1,3,5]triazine heterocyclic core is essentially planar as are both amino groups that are involved in π-electron delocalization with the triazolo[1,5-*a*][1,3,5]triazine nucleus. The methyl groups of the dimethyl­amino fragment are involved in the formation of weak intra­molecular C—H⋯N hydrogen bonds with the N atoms of the heterocyclic system. The crystal packing is stabilized by inter­molecular N—H⋯N hydrogen bonds between the triazolo[1,5-*a*][1,3,5]triazine mol­ecules. The methanol solvent mol­ecule also participates in the formation of the crystal structure *via* inter­molecular O—H⋯N, N—H⋯O and weak C—H⋯O hydrogen bonds, linking the layers of triazolo[1,5-*a*][1,3,5]triazine mol­ecules.

## Related literature

The 1,2,4-triazolo[1,5-*a*][1,3,5]triazine (5-aza­purine) heterocyclic system has been reviewed by Dolzhenko *et al.* (2006 ▶). For investigations on 5,7-diamino-1,2,4-triazolo[1,5-*a*][1,3,5]triazines, see Dolzhenko *et al.* (2007 ▶). For a similar structure, see: Gilardi (1973 ▶). For related literature, see: Dolzhenko *et al.* (2008 ▶)
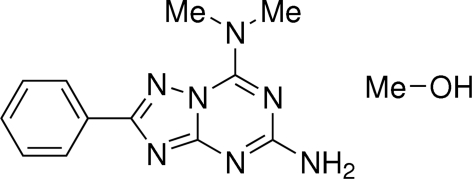


## Experimental

### 

#### Crystal data

C_12_H_13_N_7_·CH_4_O
*M* *_r_* = 287.34
Triclinic, 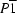

*a* = 6.9963 (5) Å
*b* = 8.0435 (5) Å
*c* = 13.0942 (9) Å
α = 93.493 (1)°
β = 93.972 (1)°
γ = 102.883 (1)°
*V* = 714.39 (8) Å^3^
*Z* = 2
Mo *K*α radiation
μ = 0.09 mm^−1^
*T* = 223 (2) K
0.74 × 0.68 × 0.40 mm

#### Data collection

Bruker SMART APEX CCD diffractometer
Absorption correction: multi-scan (*SADABS*; Sheldrick, 2001 ▶) *T*_min_ = 0.935, *T*_max_ = 0.964
9183 measured reflections
3256 independent reflections
2870 reflections with *I* > 2σ(*I*)
*R*_int_ = 0.023

#### Refinement

*R*[*F* ^2^ > 2σ(*F* ^2^)] = 0.047
*wR*(*F* ^2^) = 0.139
*S* = 1.07
3256 reflections
205 parameters
H atoms treated by a mixture of independent and constrained refinement
Δρ_max_ = 0.26 e Å^−3^
Δρ_min_ = −0.23 e Å^−3^

### 

Data collection: *SMART* (Bruker, 2001 ▶); cell refinement: *SAINT* (Bruker, 2001 ▶); data reduction: *SAINT*; program(s) used to solve structure: *SHELXS97* (Sheldrick, 2008 ▶); program(s) used to refine structure: *SHELXL97* (Sheldrick, 2008 ▶); molecular graphics: *SHELXTL* (Sheldrick, 2008 ▶); software used to prepare material for publication: *SHELXTL*.

## Supplementary Material

Crystal structure: contains datablocks I, global. DOI: 10.1107/S1600536808030481/fb2109sup1.cif

Structure factors: contains datablocks I. DOI: 10.1107/S1600536808030481/fb2109Isup2.hkl

Additional supplementary materials:  crystallographic information; 3D view; checkCIF report

## Figures and Tables

**Table 1:** Hydrogen-bond geometry (Å, °)

*D*—H⋯*A*	*D*—H	H⋯*A*	*D*⋯*A*	*D*—H⋯*A*
O1*S*—H1*S*⋯N1	0.92 (2)	1.97 (2)	2.8861 (16)	172.8 (18)
N6—H6N*B*⋯N4^i^	0.86 (2)	2.11 (2)	2.9679 (17)	178.8 (18)
N6—H6N*A*⋯O1*S*^i^	0.89 (2)	2.398 (19)	3.0280 (18)	128.3 (16)
C6—H6*C*⋯N2	0.97	2.08	2.8753 (18)	138
C6—H6*C*⋯N3	0.97	2.54	2.9484 (17)	105
C7—H7*A*⋯N5	0.97	2.22	2.6788 (18)	108
C7—H7*C*⋯O1*S*^ii^	0.97	2.48	3.4438 (19)	176

Symmetry codes: (i) 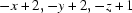; (ii) 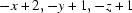.

**Fig. 1.**
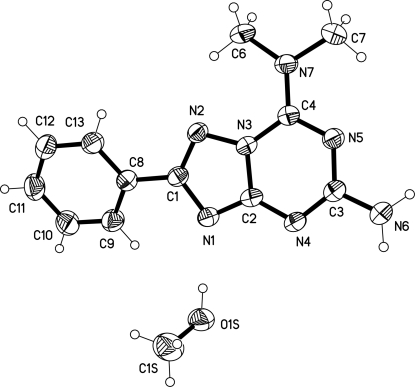
The molecular structure of the title molecules with the atomic numbering scheme. The displacement ellipsoids are drawn at the 50% probability level.

**Fig. 2.**
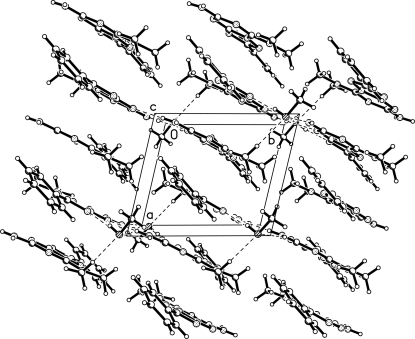
Molecular packing viewed along the axis c.

## supplementary crystallographic information

### Comment

1,2,4-Triazolo[1,5-*a*][1,3,5]triazine system is known as 5-aza-isostere of the purine core. Compounds based on this skeleton have been shown to possess a wide range of biological activities (Dolzhenko *et al.*, 2006). In continuation of our investigations on 5,7-diamino-1,2,4-triazolo[1,5-*a*][1,3,5]triazines (Dolzhenko *et al.*, 2007), we report herein the structural study of 7-dimethylamino-2-phenyl-1,2,4-triazolo[1,5-*a*][1,3,5]triazin-5-amine (Fig. 1).

The fused triazine and triazole rings are located practically in the same plane (the angle between the mean planes of these rings makes 1.66 (4)°). The phenyl ring makes a dihedral angle of 22.70 (5)° with the mean plane of the 1,2,4-triazolo[1,5-*a*][1,3,5]triazine core. Similarity of the lengths of C3—N4, C3—N5, C3—N6, C4—N3, C4—N5 and C4—N7 makes evidence for π-electron delocalization of the amino groups with the 1,2,4-triazolo[1,5-*a*][1,3,5]triazine core.

The dimethylamino group (C6—N7—C7) has an out-of-plane twist (4.3 (8)°). The nitrogen atom of dimethylamino group (N7) has a slightly pyramidal stereochemistry [C6-N7-C7 = 115.0 (1)°] and it is located 0.039 (1) Å above the C4/C6/C7 plane. These data are in good agreement with previously reported results on the similar structure of 5,7-bis(dimethylamino)-2-methylthio-1,2,4-triazolo[1,5-*a*][1,3,5]triazine (Gilardi, 1973).

The methyl groups of dimethylamino fragment are involved in the formation of weak C-H···N intramolecular hydrogen-bonds with the nitrogen atoms of the heterocyclic system (Tab. 1).

7-Dimethylamino-2-phenyl-1,2,4-triazolo[1,5-*a*][1,3,5]triazin-5-amine crystallizes together with one molecule of methanol (Fig. 1). The methanol molecule participates in the formation of the crystalline structure *via* intermolecular O-H···N, N-H···O and weak C-H···O hydrogen-bonds linking the layers of the molecules of 7-dimethylamino-2-phenyl-1,2,4-triazolo[1,5-*a*][1,3,5]triazin-5-amine (Tab. 1, Fig. 2).

### Experimental

2-Phenyl-7-trichloromethyl-1,2,4-triazolo[1,5-*a*][1,3,5]triazin-5-amine (0.66 g, 2.0 mmol) was added to cold (0–5 °C) dimethylamine (5 ml). The mixture was stirred first in ice-bath for 20 min and then for another 60 min at room temperature. Cold water (20 ml) was added and the product was filtered and recrystallized from methanol (m.p. 521 K).

### Refinement

All the hydrogen atoms could have been discerned in the difference electron density map, nevertheless, all the H atoms attached to the carbon atoms were constrained in a riding motion approximation [0.94 Å for C_aryl_-H and 0.97 Å for methyl groups; U_iso_(H) = 1.2U_eq_(C_aryl_) and U_iso_(H) =1.5U_eq_(C_methyl_)] while the hydroxyl and the amino H atoms were refined freely.

### Crystal data

**Table:**

C_12_H_13_N_7_·CH_4_O	*Z* = 2
*M**_r_* = 287.34	*F*(000) = 304
Triclinic, *P*1	*D*_x_ = 1.336 Mg m^−^^3^
Hall symbol: -P 1	Melting point: 521 K
*a* = 6.9963 (5) Å	Mo *K*α radiation, λ = 0.71073 Å
*b* = 8.0435 (5) Å	Cell parameters from 5236 reflections
*c* = 13.0942 (9) Å	θ = 2.6–27.5°
α = 93.493 (1)°	µ = 0.09 mm^−^^1^
β = 93.972 (1)°	*T* = 223 K
γ = 102.883 (1)°	Block, colourless
*V* = 714.39 (8) Å^3^	0.74 × 0.68 × 0.40 mm

### Data collection

**Table:**

Bruker SMART APEX CCD diffractometer	3256 independent reflections
Radiation source: fine-focus sealed tube	2870 reflections with *I* > 2σ(*I*)
graphite	*R*_int_ = 0.023
φ and ω scans	θ_max_ = 27.5°, θ_min_ = 2.6°
Absorption correction: multi-scan (SADABS; Sheldrick, 2001)	*h* = −9→9
*T*_min_ = 0.935, *T*_max_ = 0.964	*k* = −10→10
9183 measured reflections	*l* = −16→16

### Refinement

**Table:**

Refinement on *F*^2^	Primary atom site location: structure-invariant direct methods
Least-squares matrix: full	Secondary atom site location: difference Fourier map
*R*[*F*^2^ > 2σ(*F*^2^)] = 0.047	Hydrogen site location: difference Fourier map
*wR*(*F*^2^) = 0.139	H atoms treated by a mixture of independent and constrained refinement
*S* = 1.07	*w* = 1/[σ^2^(*F*_o_^2^) + (0.0789*P*)^2^ + 0.1582*P*] where *P* = (*F*_o_^2^ + 2*F*_c_^2^)/3
3256 reflections	(Δ/σ)_max_ < 0.001
205 parameters	Δρ_max_ = 0.26 e Å^−^^3^
0 restraints	Δρ_min_ = −0.23 e Å^−^^3^
53 constraints	

### Special details

**Table:**

Geometry. All e.s.d.'s (except the e.s.d. in the dihedral angle between two l.s. planes) are estimated using the full covariance matrix. The cell e.s.d.'s are taken into account individually in the estimation of e.s.d.'s in distances, angles and torsion angles; correlations between e.s.d.'s in cell parameters are only used when they are defined by crystal symmetry. An approximate (isotropic) treatment of cell e.s.d.'s is used for estimating e.s.d.'s involving l.s. planes.
Refinement. Refinement of *F*^2^ against ALL reflections. The weighted *R*-factor *wR* and goodness of fit *S* are based on *F*^2^, conventional *R*-factors *R* are based on *F*, with *F* set to zero for negative *F*^2^. The threshold expression of *F*^2^ > σ(*F*^2^) is used only for calculating *R*-factors(gt) *etc*. and is not relevant to the choice of reflections for refinement. *R*-factors based on *F*^2^ are statistically about twice as large as those based on *F*, and *R*- factors based on ALL data will be even larger.

### Fractional atomic coordinates and isotropic or equivalent isotropic displacement parameters (Å^2^)

**Table:**

	*x*	*y*	*z*	*U*_iso_*/*U*_eq_	
N1	0.81509 (16)	0.63570 (13)	0.67882 (8)	0.0325 (3)	
N2	0.66405 (16)	0.37327 (13)	0.60113 (8)	0.0322 (3)	
N3	0.73812 (15)	0.48432 (13)	0.52921 (8)	0.0296 (2)	
N4	0.90611 (16)	0.77645 (13)	0.52784 (9)	0.0343 (3)	
N5	0.81734 (16)	0.59004 (13)	0.37296 (8)	0.0327 (3)	
N6	0.9623 (2)	0.87296 (16)	0.36975 (11)	0.0432 (3)	
H6NA	0.954 (3)	0.859 (2)	0.3018 (16)	0.053 (5)*	
H6NB	1.001 (3)	0.975 (3)	0.3987 (15)	0.055 (5)*	
N7	0.65525 (16)	0.30863 (13)	0.37239 (8)	0.0343 (3)	
C1	0.71566 (17)	0.47092 (16)	0.68750 (10)	0.0309 (3)	
C2	0.82710 (17)	0.64251 (15)	0.57841 (10)	0.0304 (3)	
C3	0.89340 (18)	0.74312 (16)	0.42567 (10)	0.0330 (3)	
C4	0.73645 (17)	0.45873 (15)	0.42375 (10)	0.0291 (3)	
C6	0.5786 (2)	0.14747 (17)	0.41673 (12)	0.0439 (3)	
H6A	0.6609	0.0681	0.4018	0.066*	
H6B	0.4452	0.0991	0.3874	0.066*	
H6C	0.5789	0.1681	0.4905	0.066*	
C7	0.6562 (2)	0.29631 (19)	0.26060 (11)	0.0428 (3)	
H7A	0.6950	0.4101	0.2371	0.064*	
H7B	0.5255	0.2413	0.2299	0.064*	
H7C	0.7489	0.2293	0.2406	0.064*	
C8	0.66609 (18)	0.40434 (16)	0.78684 (10)	0.0333 (3)	
C9	0.7706 (2)	0.4838 (2)	0.87779 (11)	0.0431 (3)	
H9	0.8728	0.5815	0.8760	0.052*	
C10	0.7244 (3)	0.4193 (2)	0.97062 (12)	0.0517 (4)	
H10	0.7960	0.4728	1.0318	0.062*	
C11	0.5733 (3)	0.2762 (2)	0.97403 (12)	0.0512 (4)	
H11	0.5433	0.2323	1.0374	0.061*	
C12	0.4663 (2)	0.1978 (2)	0.88451 (12)	0.0456 (4)	
H12	0.3624	0.1016	0.8870	0.055*	
C13	0.5127 (2)	0.26140 (17)	0.79114 (11)	0.0386 (3)	
H13	0.4402	0.2078	0.7302	0.046*	
O1S	1.00567 (17)	0.92566 (15)	0.81876 (10)	0.0530 (3)	
H1S	0.936 (3)	0.838 (3)	0.7737 (16)	0.061 (6)*	
C1S	0.8695 (3)	0.9935 (3)	0.86937 (19)	0.0739 (6)	
H1S1	0.8051	0.9119	0.9148	0.111*	
H1S2	0.7720	1.0174	0.8195	0.111*	
H1S3	0.9360	1.0986	0.9092	0.111*	

### Atomic displacement parameters (Å^2^)

**Table:**

	*U*^11^	*U*^22^	*U*^33^	*U*^12^	*U*^13^	*U*^23^
N1	0.0333 (5)	0.0275 (5)	0.0349 (6)	0.0040 (4)	0.0007 (4)	0.0013 (4)
N2	0.0341 (5)	0.0271 (5)	0.0341 (5)	0.0038 (4)	0.0024 (4)	0.0047 (4)
N3	0.0296 (5)	0.0238 (5)	0.0336 (5)	0.0028 (4)	0.0011 (4)	0.0019 (4)
N4	0.0364 (6)	0.0256 (5)	0.0383 (6)	0.0017 (4)	0.0027 (4)	0.0021 (4)
N5	0.0333 (5)	0.0287 (5)	0.0353 (6)	0.0054 (4)	0.0039 (4)	0.0019 (4)
N6	0.0550 (8)	0.0304 (6)	0.0409 (7)	0.0007 (5)	0.0081 (5)	0.0054 (5)
N7	0.0370 (6)	0.0277 (5)	0.0355 (6)	0.0036 (4)	0.0005 (4)	−0.0014 (4)
C1	0.0279 (6)	0.0288 (6)	0.0356 (6)	0.0056 (4)	0.0009 (5)	0.0032 (5)
C2	0.0279 (6)	0.0249 (6)	0.0370 (6)	0.0048 (4)	−0.0005 (5)	−0.0006 (5)
C3	0.0302 (6)	0.0286 (6)	0.0402 (7)	0.0059 (5)	0.0042 (5)	0.0041 (5)
C4	0.0253 (5)	0.0280 (6)	0.0339 (6)	0.0068 (4)	0.0009 (4)	0.0008 (5)
C6	0.0527 (8)	0.0260 (6)	0.0473 (8)	−0.0013 (6)	0.0026 (6)	−0.0023 (5)
C7	0.0503 (8)	0.0391 (7)	0.0357 (7)	0.0062 (6)	0.0021 (6)	−0.0066 (5)
C8	0.0330 (6)	0.0326 (6)	0.0348 (7)	0.0081 (5)	0.0020 (5)	0.0042 (5)
C9	0.0414 (7)	0.0436 (7)	0.0394 (7)	0.0010 (6)	−0.0008 (6)	0.0031 (6)
C10	0.0558 (9)	0.0599 (10)	0.0340 (7)	0.0036 (7)	−0.0015 (6)	0.0021 (6)
C11	0.0560 (9)	0.0599 (10)	0.0368 (7)	0.0074 (7)	0.0106 (6)	0.0109 (7)
C12	0.0446 (8)	0.0442 (8)	0.0462 (8)	0.0029 (6)	0.0098 (6)	0.0095 (6)
C13	0.0394 (7)	0.0355 (7)	0.0388 (7)	0.0046 (5)	0.0017 (5)	0.0034 (5)
O1S	0.0485 (6)	0.0470 (6)	0.0569 (7)	−0.0004 (5)	0.0067 (5)	−0.0090 (5)
C1S	0.0679 (12)	0.0663 (12)	0.0864 (14)	0.0126 (10)	0.0233 (11)	−0.0111 (11)

### Geometric parameters (Å, °)

**Table:**

N1—C2	1.3269 (17)	C7—H7A	0.9700
N1—C1	1.3681 (16)	C7—H7B	0.9700
N2—C1	1.3172 (17)	C7—H7C	0.9700
N2—N3	1.3846 (14)	C8—C9	1.393 (2)
N3—C4	1.3826 (16)	C8—C13	1.3939 (19)
N3—C2	1.3830 (15)	C9—C10	1.381 (2)
N4—C2	1.3330 (16)	C9—H9	0.9400
N4—C3	1.3412 (18)	C10—C11	1.384 (2)
N5—C4	1.3226 (16)	C10—H10	0.9400
N5—C3	1.3524 (16)	C11—C12	1.382 (2)
N6—C3	1.3330 (17)	C11—H11	0.9400
N6—H6NA	0.89 (2)	C12—C13	1.385 (2)
N6—H6NB	0.86 (2)	C12—H12	0.9400
N7—C4	1.3323 (16)	C13—H13	0.9400
N7—C6	1.4594 (17)	O1S—C1S	1.386 (2)
N7—C7	1.4617 (18)	O1S—H1S	0.92 (2)
C1—C8	1.4709 (18)	C1S—H1S1	0.9700
C6—H6A	0.9700	C1S—H1S2	0.9700
C6—H6B	0.9700	C1S—H1S3	0.9700
C6—H6C	0.9700		
			
C2—N1—C1	103.31 (10)	N7—C7—H7A	109.5
C1—N2—N3	101.79 (10)	N7—C7—H7B	109.5
C4—N3—C2	119.85 (11)	H7A—C7—H7B	109.5
C4—N3—N2	130.72 (10)	N7—C7—H7C	109.5
C2—N3—N2	109.43 (10)	H7A—C7—H7C	109.5
C2—N4—C3	113.98 (11)	H7B—C7—H7C	109.5
C4—N5—C3	118.92 (11)	C9—C8—C13	119.13 (13)
C3—N6—H6NA	121.0 (13)	C9—C8—C1	120.54 (12)
C3—N6—H6NB	120.1 (13)	C13—C8—C1	120.33 (12)
H6NA—N6—H6NB	118.3 (18)	C10—C9—C8	120.14 (14)
C4—N7—C6	126.56 (12)	C10—C9—H9	119.9
C4—N7—C7	118.21 (11)	C8—C9—H9	119.9
C6—N7—C7	115.01 (11)	C9—C10—C11	120.31 (15)
N2—C1—N1	116.27 (11)	C9—C10—H10	119.8
N2—C1—C8	121.01 (11)	C11—C10—H10	119.8
N1—C1—C8	122.72 (12)	C12—C11—C10	120.14 (14)
N1—C2—N4	128.12 (11)	C12—C11—H11	119.9
N1—C2—N3	109.21 (11)	C10—C11—H11	119.9
N4—C2—N3	122.66 (12)	C11—C12—C13	119.79 (14)
N6—C3—N4	117.28 (12)	C11—C12—H12	120.1
N6—C3—N5	116.20 (13)	C13—C12—H12	120.1
N4—C3—N5	126.52 (12)	C12—C13—C8	120.48 (13)
N5—C4—N7	119.54 (12)	C12—C13—H13	119.8
N5—C4—N3	117.96 (11)	C8—C13—H13	119.8
N7—C4—N3	122.50 (12)	C1S—O1S—H1S	107.0 (13)
N7—C6—H6A	109.5	O1S—C1S—H1S1	109.5
N7—C6—H6B	109.5	O1S—C1S—H1S2	109.5
H6A—C6—H6B	109.5	H1S1—C1S—H1S2	109.5
N7—C6—H6C	109.5	O1S—C1S—H1S3	109.5
H6A—C6—H6C	109.5	H1S1—C1S—H1S3	109.5
H6B—C6—H6C	109.5	H1S2—C1S—H1S3	109.5
			
C1—N2—N3—C4	178.41 (12)	C6—N7—C4—N5	173.18 (12)
C1—N2—N3—C2	−0.83 (12)	C7—N7—C4—N5	−1.02 (18)
N3—N2—C1—N1	0.51 (14)	C6—N7—C4—N3	−7.5 (2)
N3—N2—C1—C8	179.98 (10)	C7—N7—C4—N3	178.25 (11)
C2—N1—C1—N2	0.03 (14)	C2—N3—C4—N5	−1.60 (17)
C2—N1—C1—C8	−179.44 (11)	N2—N3—C4—N5	179.22 (11)
C1—N1—C2—N4	178.09 (12)	C2—N3—C4—N7	179.11 (11)
C1—N1—C2—N3	−0.57 (13)	N2—N3—C4—N7	−0.1 (2)
C3—N4—C2—N1	−179.24 (12)	N2—C1—C8—C9	159.09 (13)
C3—N4—C2—N3	−0.74 (18)	N1—C1—C8—C9	−21.47 (19)
C4—N3—C2—N1	−178.42 (10)	N2—C1—C8—C13	−21.37 (18)
N2—N3—C2—N1	0.92 (13)	N1—C1—C8—C13	158.07 (12)
C4—N3—C2—N4	2.83 (18)	C13—C8—C9—C10	1.2 (2)
N2—N3—C2—N4	−177.83 (11)	C1—C8—C9—C10	−179.30 (14)
C2—N4—C3—N6	177.38 (11)	C8—C9—C10—C11	−0.5 (3)
C2—N4—C3—N5	−2.68 (19)	C9—C10—C11—C12	−0.6 (3)
C4—N5—C3—N6	−176.15 (11)	C10—C11—C12—C13	0.9 (3)
C4—N5—C3—N4	3.9 (2)	C11—C12—C13—C8	−0.2 (2)
C3—N5—C4—N7	177.83 (11)	C9—C8—C13—C12	−0.8 (2)
C3—N5—C4—N3	−1.48 (17)	C1—C8—C13—C12	179.66 (12)

### Hydrogen-bond geometry (Å, °)

**Table:**

*D*—H···*A*	*D*—H	H···*A*	*D*···*A*	*D*—H···*A*
O1S—H1S···N1	0.92 (2)	1.97 (2)	2.8861 (16)	172.8 (18)
N6—H6NB···N4^i^	0.86 (2)	2.11 (2)	2.9679 (17)	178.8 (18)
N6—H6NA···O1S^i^	0.89 (2)	2.398 (19)	3.0280 (18)	128.3 (16)
C6—H6C···N2	0.97	2.08	2.8753 (18)	138.
C6—H6C···N3	0.97	2.54	2.9484 (17)	105.
C7—H7A···N5	0.97	2.22	2.6788 (18)	108.
C7—H7C···O1S^ii^	0.97	2.48	3.4438 (19)	176.

Symmetry codes: (i) −*x*+2, −*y*+2, −*z*+1; (ii) −*x*+2, −*y*+1, −*z*+1.
